# Evaluation of Open-Source Ciliary Analysis Software in Primary Ciliary Dyskinesia: A Comparative Assessment

**DOI:** 10.3390/diagnostics14161814

**Published:** 2024-08-20

**Authors:** Zachary J. Demetriou, José Muñiz-Hernández, Gabriel Rosario-Ortiz, Frances M. Quiñones, Gabriel Gonzalez-Diaz, Marcos J. Ramos-Benitez, Ricardo A. Mosquera, Wilfredo De Jesús-Rojas

**Affiliations:** 1 Department of Pediatrics and Basic Science, Ponce Health Sciences University, Ponce, PR 00716, USA; zdemetriou22@stu.psm.edu (Z.J.D.); grosario@psm.edu (G.R.-O.); frquinones@psm.edu (F.M.Q.); ggonzalez21@stu.psm.edu (G.G.-D.); mjramos@psm.edu (M.J.R.-B.); 2 San Juan Bautista School of Medicine, Caguas, PR 00725, USA; josemh@sanjuanbautista.edu; 3 Department of Pediatrics, McGovern Medical School, University of Texas Health Science Center at Houston, Houston, TX 77030, USA; ricardo.a.mosquera@uth.tmc.edu

**Keywords:** primary ciliary dyskinesia, ciliary beat frequency, high-speed video microscopy analysis, Cilialyzer, CiliarMove

## Abstract

Primary Ciliary Dyskinesia (PCD) is a rare genetic disorder characterized by alterations in motile cilia function. The diagnosis of PCD is challenging due to the lack of standardized methods in clinical practice. High-speed video microscopy analysis (HSVA) directly evaluates ciliary beat frequency (CBF) in PCD. Recently, open-source ciliary analysis software applications have shown promise in measuring CBF accurately. However, there is limited knowledge about the performance of different software applications, creating a gap in understanding their comparative effectiveness in measuring CBF in PCD. We compared two open-source software applications, CiliarMove (v219) and Cilialyzer (v1.2.1-b3098cb), against the manual count method. We used high-speed videos of nasal ciliary brush samples from PCD *RSPH4A*-positive (PCD (*RSPH4A*)) patients and healthy controls. All three methods showed lower median CBF values for patients with PCD (*RSPH4A*) than in healthy controls. CiliarMove and Cilialyzer identified lower CBF in patients with PCD (*RSPH4A*), similarly to the manual count. Cilialyzer, CiliarMove, and manual count methods demonstrated statistical significance (*p*-value < 0.0001) in the difference of median CBF values between patients with PCD (*RSPH4A*) and healthy controls. Correlation coefficients between the manual count values against both software methods demonstrated positive linear relationships. These findings support the utility of open-source software-based analysis tools. Further studies are needed to validate these findings with other genetic variants and identify the optimal software for accurate CBF measurement in patients with PCD.

## 1. Introduction

Primary Ciliary Dyskinesia (PCD) is a rare autosomal recessive genetic disease with an estimated prevalence of approximately 1:7500 to 1:15,000 individuals [1,2]. The functional role of the motile cilia is exemplified by the spectrum of phenotypical manifestations seen in PCD [3]. The clinical observations of PCD ultimately stem from the functional impairment of cilia, leading to ciliary dyskinesia [4]. Common clinical features include a year-round wet cough, neonatal respiratory distress, recurrent respiratory tract infections leading to bronchiectasis, persistent rhinosinusitis, recurrent otitis media, infertility, as well as organ laterality defects depending on the affected gene [5]. Despite the early onset of symptoms, PCD diagnosis is often delayed, resulting in underdiagnosis and missed opportunities for early intervention [6]. This delay can be partially attributed to the spectrum of clinical presentations of the disorder, and the concomitant presence of other chronic respiratory diseases, posing challenges in accurate identification and leading to potential diagnostic gaps [7].

Presently, there is no gold standard test for PCD [8]. ATS and ERS guidelines agree that PCD diagnosis can be achieved with confirmation of hallmark ultrastructural defects or a genetic test. However, on their own, neither Transmission Electron Microscopy (TEM) or genetic testing have good sensitivity [9]. For example, it is estimated that Transmission Electron Microscopy use in identification of hallmark ciliary ultrastructure is non-diagnostic in 30% of PCD cases [10]. In addition, genetic sequencing testing is estimated to be non-diagnosable in 20–30% of cases [11]. Moreover, issues arise in following an algorithm for an accurate diagnosis due to several factors, including limited access and expertise required for recommended PCD diagnostic tests, especially in underdeveloped countries [12]. Specialized PCD diagnostic centers in the United States and Europe employ algorithms that incorporate multiple diagnostic tools, such as measuring nasal nitric oxide (nNO), genetic sequencing, immunofluorescence, and high-speed video microscopy analysis (HSVA) [13,14]. However, the availability of these diagnostic tools is limited worldwide, and their operation requires specialized expertise [15]. To date, diagnosing PCD remains a complex task that requires the integration of multiple modalities and a collaborative effort from a multidisciplinary team [13].

Among the available diagnostic tools, HSVA is the only method that enables direct observation of ciliary motion and assessment of ciliary function [5]. In HSVA, the ciliary beat frequency (CBF) and ciliary beat pattern (CBP) are key observable variables for PCD evaluation [16]. HSVA and CBF measurement are key diagnostic tools, but manual counting is tedious and subjective [17,18]. Software tools like CiliarMove (v219) and Cilialyzer (v1.2.1-b3098cb) have emerged to automate CBF analysis from HSVA [17,19,20,21]. The first commercially available software, the Sisson–Ammons Video Analysis (SAVA) system, was developed in 2003 [18]. The development of freely available open-source software followed, which notably included the software named CiliaFA (v1) in 2012, but more recently, CiliarMove in 2021, and Cilialyzer in 2022 [17,20,21]. All the software applications mentioned above have individually demonstrated the capability to accurately calculate CBF, aligning with manually determined values [17,18,20,21]. Among the available open-source software platforms, CiliarMove and Cilialyzer have been validated independently in recent literature for their ability to perform such analyses [20,21]. Table 1 provides a comparative analysis of the two most recent open-source software tools, CiliarMove and Cilialyzer.

Despite establishing clinical utility, knowledge regarding performance analysis across software platforms is limited. When considering the origin of such software, it is reasonable to expect further developments and improvements. A software automating analysis sounds promising when coupled with a disorder requiring diagnostic standardization. Thus, as the technology develops, analysis across software platforms could provide useful information aiding HSVA standardization. Our study presents a comparison of the ability of manual count, Cilialyzer, and CiliarMove open-source software to compute CBF from healthy control and PCD patients with a confirmed diagnosis of the same *RSPH4A* (c.921+3_921+6delAAGT (intronic)) founder mutation [22].

## 2. Materials and Methods

### 2.1. Subjects

The study included 12 patients with confirmed PCD with bi-allelic pathogenic *RSPH4A* (c.921+3_921+6del (intronic)) founder mutation (*n* = 12) and six healthy controls (*n* = 6). The healthy controls were not smokers, and none exhibited any clinical symptoms associated with PCD, asthma, or chronic respiratory illness as per past medical history. At the time of nasal biopsy, all subjects from the PCD (*RSPH4A)* cohort and healthy controls were at their baseline status of health for more than two weeks, free of any upper respiratory tract infection.

### 2.2. Sample Collection and Preparation

Nasal ciliated epithelial samples were collected with cytology brushing (Puritan Sterile Cytology Brushes). The cytology brush was inserted briefly in one nostril and gently moved into and out of the inferior nasal turbinate ten times while performing internal rotation of the brush and pressing slightly on the lateral wall of the inferior turbinate [23]. Once brushing was completed, sample preparation began immediately. The cytology brush was twisted in 3 mL of PneumaCult™-Ex Plus Medium to remove any tissue that remained adherent to the bristles. The sample was required to be placed in a sufficient volume of media, permitting full immersion of the brush. Samples were washed in 5 mL of D-PBS without calcium and magnesium to assist in removing mucus and debris. The sample was then centrifuged at 400× *g* for seven minutes in order to pellet the epithelial cells. The nasal brushings were then resuspended in 500 μL of PneumaCult™-Ex Plus Medium and incubated at 37 °C for 30 min prior to HSVA at 500 fps under 60× magnification. After this period, 100 μL of the cell suspension was plated in a glass slide for analysis. A total of one biopsy per patient with PCD founder mutation or healthy control was collected.

### 2.3. Highspeed Video Microscopy Analysis (HSVA)

The observation of samples was conducted using the Nikon Eclipse Ti2 inverted microscope with a long working distance 60× objective lens. An AOS PROMON U750 monochrome high-speed camera was attached to the microscope to capture high-speed video recordings. The camera had a light sensitivity grade of ISO 3600 and a sensor size of 4.8 μm pixel. This setup permitted recording samples at a frame rate of 500 frames per second (fps). The AOS Imaging Software Version 4 was used to process the footage. A microscope air table was employed in conjunction with the HSVA to minimize movement during the recording. The optimal resolution setting for HSVA recorded at 500 fps was recommended by the AOS software in the camera suite and set at 880 × 637 pixels. Following the guidelines of the European Respiratory Society (ERS) [13], 2200 frames were recorded for five seconds at the specified resolution and under 60×. The recorded HSVA footage was saved as unprocessed image data (RAW) files. During the analysis, the region of interest (ROI) was selected only if it was void of mucus or debris on the surface of the cell cluster. To ensure the footage was representative of the ciliary population, an ROI was selected only if 10–15 equally distant adjacent ciliated cells were present. One ROI per sample was selected. A thermal plate (F/TS2R & TI2 Stage) for Nikon Eclipse Ti2 Inverted microscope was used to maintain extended observation of motile cilia at 37 °C to minimize variability in CBF. The cell clusters that were intact were chosen over single cells to obtain a more representative sample [24]. All the HSVA was completed in less than 30 min per sample. All three analysis procedures included the same sample videos.

### 2.4. Manual Method for CBF Count

CBF was counted manually from 10 beat cycles, which was followed by CiliarMove and Cilialyzer in a standardized workflow. The manual method for CBF counting was performed as previously described in the literature using the following formula: manual CBF (Hz) = (fps/number of frames elapsed for 10 full ciliary beats) × 10 (conversion per beat cycle) [17,21]. When performing the manual CBF count, we paused the recordings at a point where the cilia were closest to the maximal bend. We adjusted the footage frame by frame until most of the cilia were fully bent; at this point, we marked the starting frame for reference. We counted the number of full ciliary beats, including the forward and backstroke, from the starting to the end frame. We then recorded the number of frames that elapsed while observing ten complete ciliary beats for the given cycle.

### 2.5. Software Analysis

The Cilialyzer (v1.2.1-b3098cb) and CiliarMove (v219) software were applied to the same set of recordings provided by the patients with PCD (*RSPH4A*) and healthy controls. A reference picture of the ROI marked with a cursor was applied to ensure consistent analysis of the same ROI. For CBF computation using CiliarMove, the RAW format video files required one conversion step to be transformed into the tag image file format (TIFF) sequence file. After the file conversion, the image sequence was uploaded for analysis. Before analyzing the frequency, the ROI was manually selected using the cropping function. For Cilialyzer, the RAW format video files were converted into sequence images in portable network graphics (PNG) format and pre-processed as stated by the Cilialyzer manual [20]. The pre-processing methods included initial manual image rotation, selecting ROI, image stabilization, cropping margins, and motion extraction. The CBF was then calculated by both CiliarMove and Cilialyzer, with the results displayed on a frequency distribution histogram and a power spectrum graph, respectively. These steps were followed to ensure accurate and consistent analysis of the CBF using Cilialyzer and CiliarMove for the respective software platforms.

### 2.6. Statistical Analysis

Descriptive statistics, including median and interquartile ranges (IQRs), were calculated to summarize the data, providing measures of central tendency and dispersion. The normality of the data was assessed using the Kolmogorov–Smirnov test. The healthy control CBF value data sets were normally distributed across all three methods of analysis. The CBF value data sets for the PCD (*RSPH4A*) cohort were normally distributed across the manual count method and Cilialyzer, but not CiliarMove. Parametric data of CBF measurements between the PCD (*RSPH4A*) and healthy control groups were compared using the Welch’s t test. Non-parametric data were compared using one-sample Wilcoxon test comparing CBF between PCD (*RSPH4A*) and control for each method. A significance level of α = 0.05 was set to determine statistical significance, and all reported *p*-values were two-tailed. A *p*-value less than 0.05 indicated a statistically significant difference in CBF median measurements between the two groups. Based on the finite population correction for the available 25 patients with PCD (*RSPH4A*) in Puerto Rico with the founder genetic mutation, the sample size calculations indicated that 12 participants in the PCD (*RSPH4A*) group and six healthy controls were sufficient to detect significant differences in CBF between groups. The adjusted sample sizes ensured adequate statistical power to validate the performance of CiliarMove and Cilialyzer software compared to the manual count method. These sample sizes were determined to be adequate for detecting meaningful differences in CBF with a high degree of confidence (*p*-value < 0.0001), ensuring the robustness and reliability of our findings. A correlation coefficient was also created between the manual count and both software for both cohorts. For the parametric data, a Pearson’s correlation coefficient was calculated, and for the nonparametric data, a Spearman’s correlation coefficient was calculated. All correlation coefficient analyses were deemed to be statistically significant (*p* value < 0.0001). All statistical analyses were performed using the statistical software package GraphPad Prism version 10.0.0 for Windows, developed by GraphPad Software (v. 10.3.0), San Diego, CA, USA [www.graphpad.com].

## 3. Results

This study included 115 high-speed microscopy recordings from twelve patients (*n* = 12) with PCD and 60 videos from six healthy controls (*n* = 6). Each recording underwent analysis using three different CBF computational methods, resulting in 175 CBF observation points. Median CBF was 9.2 Hz in patients with PCD (*RSPH4A*) versus 11.1 Hz in the healthy control by manual counting. CiliarMove measured median CBF of 8.8 Hz and 10.7 Hz in PCD (*RSPH4A*) and healthy control groups, respectively. Cilialyzer showed the largest difference, with medians of 8.7 Hz for PCD (*RSPH4A*) and 10.8 Hz in the healthy control group. The median difference in CBF between PCD (*RSPH4A*) and healthy control was 1.9 Hz for manual count, 1.9 Hz for CiliarMove, and 2.1 Hz for Cilialyzer (Table 2).

**Figure 1:**
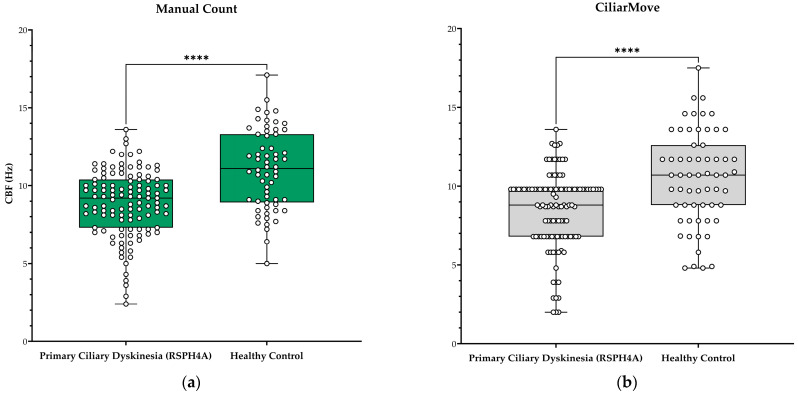

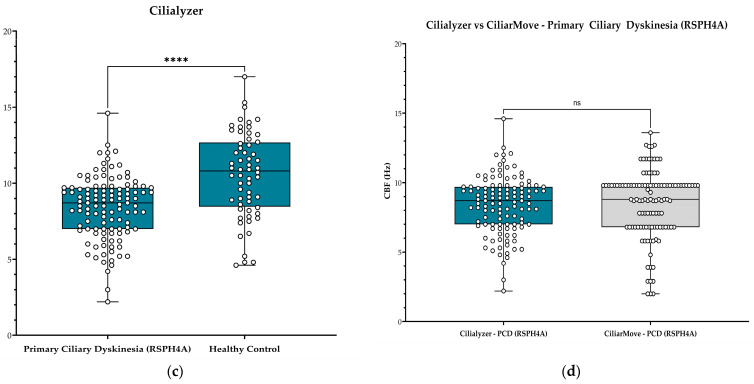
CBF values in hertz (Hz) from patients with Primary Ciliary Dyskinesia (PCD) *RSPH4A* (c.921+3_921+6del (intronic) founder mutations and healthy controls. Box and whisker plot represented with median and IQR values: (**a**) manual count CBF values from PCD (*RSPH4A*) cohort and healthy control, (**b**) CiliarMove, and (**c**) Cilialyzer. (**d**) A comparison between Cilialyzer and CiliarMove using recordings from patients with PCD (*RSPH4A*). Level of statistical significance: * *p* < 0.05; ** *p* < 0.01; *** *p* < 0.001; **** *p* < 0.0001 and ns = not significant.

**Figure 2:**
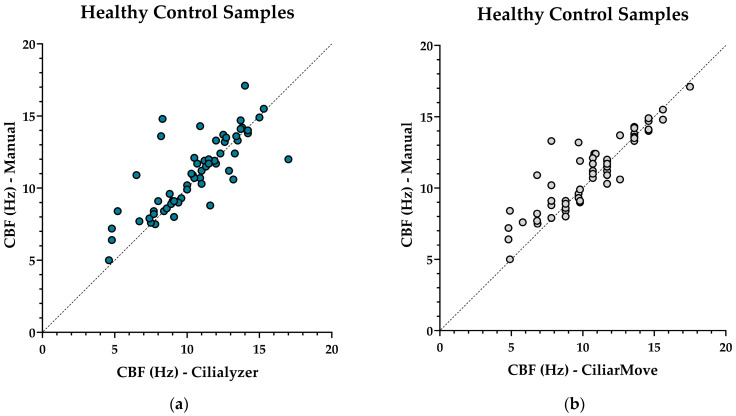

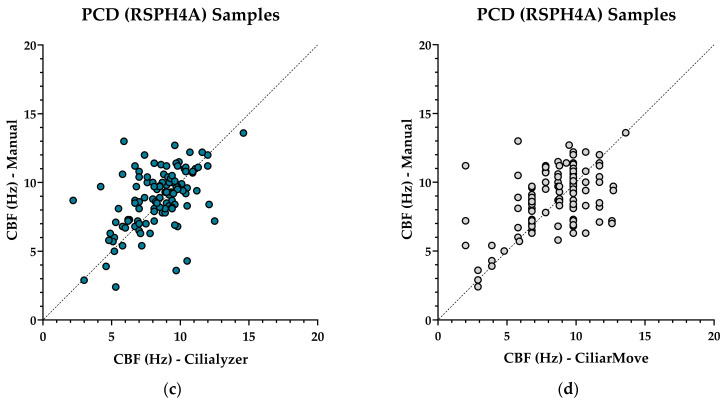
CBF values in hertz (Hz) from patients with Primary Ciliary Dyskinesia (PCD) *RSPH4A* (c.921+3_921+6del (intronic) founder mutations and healthy controls. Correlation coefficient: (**a**) healthy control samples: manual count, and Cilialyzer; (**b**) healthy control samples: manual count and CiliarMove; (**c**) PCD (*RSPH4A*): manual count and Cilialyzer; (**d**) PCD (*RSPH4A*): manual Count and CiliarMove.

All methods gave significantly lower CBF values in PCD patients compared to the healthy control (*p*-value < 0.001). The healthy control cohort measured with Cilialyzer had a strong positive linear relationship, with an r value of 0.7964, while the CiliarMove cohort had a stronger positive linear relationship, with an r value of 0.8947. In the PCD (*RSPH4A*) cohort, Cilialyzer had a strong positive linear relationship, with an r value of 0.5206, while CiliarMove had a moderate positive linear relationship, with an r value of 0.3830. Cilialyzer, therefore, maintained a strong positive linear relationship in both cohorts, while CiliarMove maintained a strong positive linear relationship only in the healthy control cohort (Figure 1 and Figure 2).

## 4. Discussion

This study compared the CBF between individuals diagnosed with PCD positive for *RSPH4A* (c.921+3_921+6del (intronic) founder mutations and healthy controls. We evaluated CBF data using two software tools (Cilialyzer and CiliarMove) and manual method counting. Utilizing the one-sample Wilcoxon test and Welch’s t test for statistical analysis, we identified significant differences in CBF values between the PCD (*RSPH4A*) patients and healthy controls for all three methods. Cilia samples in PCD (*RSPH4A*) exhibited a slower frequency than a significantly faster beating frequency in samples from the healthy controls. Both software types computed CBF through the power spectral density means of each pixel’s variations in light intensity, as previously demonstrated [17,21]. All CBF values obtained corresponded well with the manual count. When evaluating the performance of the two software tools, Cilialyzer had the greatest actual CBF median difference between groups. These results indicate that all methods successfully differentiated CBF between PCD patients from healthy controls.

The correlation coefficient demonstrated that Cilialyzer maintained a strong positive linear relationship in both cohorts, while CiliarMove demonstrated a strong and moderate positive linear relationship in the healthy control and PCD (*RSPH4A*) cohorts, respectively. However, in the healthy control cohort, CiliarMove did have a higher r-value when compared to Cilialyzer. Both software types analyzed the same 1000 frames of HSVA sample recording, providing a performative comparison in reference to the manual count. Using the CBF calculation formula from the papers published on both software programs [17,21], this formula permits a different number of frames elapsed to reach the 10 ciliary beats needed to calculate the CBF. Therefore, we were unable to use the same fixed number of frames elapsed used in the software analysis. Since this was not possible, we chose a standard frame count for the software analysis and focused on the comparison of the software performance to a manual reference, rather than attempting to evaluate whether manual count method or software is more effective.

Image stabilization and motion extraction features may have improved signal quantification; Schneiter et al. demonstrated the importance of image stabilization in a freshly excised group of nasal epithelial cells exhibiting a superposed quasi-periodic motion on the ciliary function within the ROI [20]. This resulted in an inaccurate reading of the average power spectral density, which compromised both the display of the CBF-bandwidth and the actual CBF peak value. The average power spectral density was corrected by applying the image stabilization feature. This feature allows Cilialyzer to remove the undesirable sample motion through the use of its Python package ’pyStackReg’ (v.0.2.7) [25], which results in the alignment of consecutive images in the sequence to remain in place. The other notable pre-processing method offered by Cilialyzer is the “motion extraction” feature, which is performed via a computational function termed “mean image subtraction”, which has been previously described in the literature [26,27]. Motion extraction removes the static background of an image, which was demonstrated to enhance the visualization of the cilia along with their movement in the selected ROI. These methods bolster the quantitative objective analysis, ensuring its correctness, and also improve the visual assessment of manual count by highlighting the observable cilia [20]. The clinical value of automated software use in CBF analysis has been supported in prior literature, demonstrating improved efficiency and decreased subjectivity with the use of Sisson–Ammons Video Analysis (SAVA) compared to the manual count method for CBF measurement [18]. Furthermore, the findings from a double-blind, placebo-controlled, multicenter trial demonstrated clinical application and validation when using CiliarMove for CBF analysis in cystic fibrosis receiving nebulized gene therapy [28].

A limitation of this study was the small sample size. Considering the relatively strict objective analytic nature of investigating software, we hoped to avoid any misleading data from a small sample size through providing a relatively stable CBF to be measured in both cohorts and an adequate amount of sample biopsies analyzed (>100) to support statistical analysis of the one-sample Wilcoxon test. Using a shared PCD founder mutation and a group of healthy control ciliated epithelial cells for analysis, we attempted to apply a consistent method to extract data. Theoretically, if both high- and low-frequency data sets were consistent, a closer measure of the ability to differentiate CBF could be analyzed. Also of note, the study was not matched for age or gender. However, age and CBF correlation appear to be a controversial subject matter as it has been noted to have been investigated in insufficient studies to confirm a correlation between the two variables. Despite this, some evidence supports that individuals aged above 40 years demonstrate a slower CBF and decreased mucociliary clearance [29,30,31,32].

Another limitation was the differences in ROIs between CBF measurement methods. Despite our efforts to obtain ROIs of relative similarity through a reference image, this was not pixel-per-pixel accurate. In addition, the different file formats used to analyze the sequences may have influenced the results. It would be interesting to perform a comparative analysis with these factors matched in a study design.

Our study did not complete a comparative software analysis with different PCD genotypes. Certain PCD genotypes are known to have relatively higher or lower CBF, and, thus, the comparative performance of Cilialyzer and CiliarMove in such cases remains unclear. In such instances, CiliarMove’s ability to measure CBF relative to the manual count method was supported in the study performed by Sampio P et al. [21], which included PCD subjects with a genetic mutation in *DNAH11* and *DNHA5* displaying a faster and slower CBF, respectively. The mean CBF value calculated from the manual count method and CiliarMove, using 10 ROIs per patient, was comparable across these three patients (manual count Hz/CiliarMove Hz: 14.30/14.34, 5.74/5.70, 5.83/5.71). Further directions should include the comparative efficacy of software analyzing different genetic PCD variants with known CBFs. Additional research on cilia analysis software should be undertaken when new developments arise; as such, feedback could be conducive to further improvements.

Freely available open-source software, such as CiliaFA, CiliarMove, and Cilialyzer, had literature published documenting their efficacy from 2012, 2021, and 2022, respectively [17,20,21]. The first two software programs were developed excluding programming that permitted methodical pre-processing ability. Within one year of CiliarMove, literature was published on Cilialyzer, which almost appears as a radically differently functioning freely available type of software. Considering the cost of commercially available software lacking Cilialyzer’s added features, the development of freely available software is well-deserving of recognition. A freely available open-source code coupled with multiple added features, from ROI correction to spatial frequency maps and particle tracking, provides a promising outlook on the capability and accessibility of future developments in such technology. However, when using HSVA, multiple parameters are assessed from the recordings, including CBF, ciliary beat pattern (CBP), and mucociliary clearance [33]. The use of CBF analysis alone in PCD diagnosis is insufficient [9]. Isolated CBF analysis in PCD diagnosis results in low sensitivity and specificity, which are partially resolved when combined with CBP, rendering higher positive and negative predictive values [34]. Therefore, it should be emphasized that open-source software with the capability of analyzing CBF, CBP, and other variables would need to be developed to automate ciliary functional analysis fully.

## 5. Conclusions

Our findings demonstrate that manual count, Cilialyzer, and CiliarMove are effective tools for evaluating CBF in PCD patients, as they could accurately identify lower CBF values compared to healthy controls. However, our results highlight the practicality of utilizing software-based analysis tools in diagnosing and assessing PCD. Each open-source platform offers a semi-automated and less time-consuming option for evaluating CBF, overcoming the subjectivity and labor-intensive nature of manual counting. The findings of this study contribute to the growing body of evidence supporting the utility of open-source ciliary analysis software in PCD evaluation and offering valuable insights for researchers and clinicians in selecting the appropriate software tool for accurate CBF measurement. However, the performance capability of open-access software in analyzing CBF for other genotypes with relatively higher or lower CBF remains unknown. Further research is warranted to validate these findings across different genetic variants, age groups, and new software platforms. Overall, this study highlights the importance of leveraging technology and software advancements to improve the diagnostic capabilities of PCD.

## Figures and Tables

**Table 1:** Comparative analysis of Cilialyzer and CiliarMove open-source software for ciliary beat frequency (CBF) analysis in primary ciliary dyskinesia (PCD).

	Cilialyzer [20]	CiliarMove [21]
Authors	Schneiter M, et al.	Sampaio P, et al.
Year of Publication	2022	2021
Purpose	Standardized identification of impaired mucociliary activity, facilitating diagnostic testing for PCD.	Evaluating CBF to aid in the correct diagnosis of PCD.
Innovation	Provides new quantitative information for PCD diagnostics.	Provides an open-source, fast, and intuitive tool for CBF evaluation.
Main Features	Semi-automated analysis of mucociliary activity captured by HSVA.	Semi-automated evaluation of CBF using HSVA.
	Analyzes ciliated epithelial cell cluster samples, as well as intact mucociliary epithelium culture.	Analyzes CBF from respiratory epithelial cell clusters.
	Capable of analyzing CBF from HSVA at a rate of 500 frames per second as recommended by the European Respiratory Society (ERS).	
	Compute CBF within a sequence of images in greyscale by calculating the fast Fourier transform method for each pixel of a given region of interest (ROI).	
Pre-processing features Sample-type application: Brushed human nasal epithelial cells immersed in medium	Manual rotation of image	Manual selection of ROI Video visualization panelIncludes play/pause feature, adjustable frame speed, and displays size of selected ROI measured in pixels.
	Manual selection of ROI	
	Image stabilization	
	Motion extraction	
Post-processing features Sample-type application: Brushed human nasal epithelial cells immersed in medium	Power spectrum Displays CBF as the peak bandwidth of	Heat map of CBF Color gradient indicates faster vs. slower frequency
	Visual assessment Display panel includes adjustable frame speed, play/pause feature, forward/backward playback option of image sequence, zoom function, and image contrast settings.	Fast Fourier transform plot For any selected pixel
		Histogram of CBF distribution From the selected ROI
		Table display of CBF percentages From the selected ROI
Pre/post-processing features Sample-type application: Intact cultures of mucociliary epithelium	“Pre-processing” features here include allmethods as described above.	Software not programmed to analyze intact mucociliary epithelium.
	Power spectrum	
	Activity map of spatial CBF distribution	
	Frequency correlation length	
	Particle extraction	
	Particle tracking	
	Mucociliary transport speed	
Validation/testing	The computational analysis methods of Cilialyzer are demonstrated using simulated and representative sample data from clinical practice.	Correlation coefficient R^2^ = 0.9895 when comparing CiliarMove to the manual observation method. CBF values were obtained from a healthy control group, a PCD-excluded group, and a PCD-confirmed group.
Availability	Freely available under the terms of the Massachusetts Institute of Technology (MIT) license.	Open-source program, but the specific license is not mentioned.
User-friendliness	The software stands out due to its ease of customization and extension. It requires no license or virtual machine. It provides many pre-processing and replaying options, making it easy to use.	The user-friendly interface allows less-trained users to perform the evaluation of the CBF.
Cost-effectiveness	The cost-effectiveness of using the Cilialyzer for PCD diagnosis has not been explicitly discussed.	Provides cost benefits by adapting to any high-speed image setup, enabling low-budget HSVA in resource-limited settings.

**Table 2:** Number of samples analyzed per cohort, median CBF per cohort, actual median CBF difference. Statistical analysis represented by one-sample Wilcoxon test and Welch’s *t* test for each of the three CBF computation methods used.

	Manual Count		CiliarMove		Cilialyzer	
	PCD (*RSPH4A*)	HC	PCD (*RSPH4A*)	HC	PCD (*RSPH4A*)	HC
Median CBF (Hz)	9.2	11.1	8.8	10.7	8.7	10.8
Actual median difference	1.9		1.9		2.1	
*p*-value	** <0.0001		* <0.0001		** <0.0001	
Correlation coefficient r-value			0.3830	0.8947	0.5206	0.7964

* Two tailed *p*-value: calculated via one-sample Wilcoxon test, ** two tailed *p*-value: calculated via Welch’s *t* test, PCD (*RSPH4A*): Primary Ciliary Dyskinesia *RSPH4A* positive, HC: healthy controls, Hz: hertz units.

## Data Availability

All data are available upon request through the corresponding author.
